# A Multi-level Review of Engineering Ethics Education: Towards a Socio-technical Orientation of Engineering Education *for* Ethics

**DOI:** 10.1007/s11948-021-00333-6

**Published:** 2021-08-24

**Authors:** Diana Adela Martin, Eddie Conlon, Brian Bowe

**Affiliations:** 1 Philosophy and Ethics, Department IE&IS, Eindhoven University of Technology, Eindhoven, The Netherlands; 2 College of Engineering and Built Environment, Technological University Dublin, Dublin, Ireland; 3 Academic Affairs – City Campus, Technological University Dublin, Dublin, Ireland

**Keywords:** Literature review, Engineering ethics education, Implementation of ethics, Ethics instruction, Learning goals, Curricular alignment, Engineering culture, Engineering education reform, Socio-technical engineering education

## Abstract

This paper aims to review the empirical and theoretical research on engineering ethics education, by focusing on the challenges reported in the literature. The analysis is conducted at four levels of the engineering education system. First, the individual level is dedicated to findings about teaching practices reported by instructors. Second, the institutional level brings together findings about the implementation and presence of ethics within engineering programmes. Third, the level of policy situates findings about engineering ethics education in the context of accreditation. Finally, there is the level of the culture of engineering education. The multi-level analysis allows us to address some of the limitations of higher education research which tends to focus on individual actors such as instructors or remains focused on the levels of policy and practice without examining the deeper levels of paradigm and purpose guiding them. Our approach links some of the challenges of engineering ethics education with wider debates about its guiding paradigms. The main contribution of the paper is to situate the analysis of the theoretical and empirical findings reported in the literature on engineering ethics education in the context of broader discussions about the purpose of engineering education and the aims of reform programmes. We conclude by putting forward a series of recommendations for a socio-technical oriented reform of engineering education *for* ethics.

## Introduction

Ethical concerns are a contemporary addition in engineering education. The establishment of ethics as an academic subject in the engineering curriculum began in the 1970s, when research on engineering ethics started to feature in academic journals and dedicated textbooks were published (Mitcham, 2009; Weil, 1984). Traditionally, disciplines of exact sciences such as engineering were regarded as morally neutral (Roeser, 2012) or even as morally good,^1^ and hence did not require ethical instruction (Ehrlich, 2010). Consequently, the development of engineering ethics education has been slow (Mitcham, 2009; Reed et al., 2004).

The article aims to analyse the education of engineering ethics in terms of the challenges and dissatisfaction reported in the literature and link these with debates about the paradigms guiding engineering education and the purpose of reform programmes. The literature review draws inspiration from the Critical Realist focus on different levels of the engineering education system, which locates individual agents in the socio-cultural and institutional contexts in which they operate (Conlon, 2015). The failure to integrate these different levels into programs for change has been identified as a gap in engineering education research, with different research communities having focused separately on different levels (Froyd et al., 2008; Seymour, 2002). As Godfrey (2014, p. 438) points out, it is important to focus the analysis of engineering education not only on the characteristics of behaviours and practices, but also on the values, beliefs, and assumptions that underpin how these came to be, as to enable the development of reform strategies. As such, higher education research should be mindful of contextual aspects, given that reform programmes focused strictly on ‘improving’ individuals run the risk of failure when neglecting the broader context that individuals operate in (Trowler, 2008, p. 151). This can explain why engineering education reform has a relatively long but slow history (Heywood, 2005).

During the past decades, numerous challenges and an overall dissatisfaction with the state and status of engineering ethics education have been highlighted. The challenges revealed by empirical and conceptual research are preponderantly of an individual manner, pertaining to the instructors’ struggle to make sense of the variety of theoretical frameworks, learning goals, teaching activities and assessment methods, as to ensure their alignment (Keefer et al., 2014). This challenge is compounded by the engineering instructors’ low familiarity with ethics and their access to institutional support, CPD programmes or teaching resources. Several challenges of an institutional nature have also been reported. These are related to the unsystematic implementation of ethics (Colby & Sullivan, 2008; Barry & Ohland, 2012; Flynn & Barry, 2010; Polmear et al., 2018), as well as the low weight given to ethics (Barry & Ohland, 2012; Colby & Sullivan, 2008; Monteiro et al., 2016). There are also challenges related to the cultural milieu of engineering education that was formative for the current generation of engineering academics (Jamison et al., 2014), and which in turn impacts the instructors and students’ engagement with ethics (Barry & Herkert, 2014; Besterfield-Sacre et al., 2000; Cech, 2014; Sheppard et al., 2009). These challenges point to the complexity behind the implementation and teaching of engineering ethics, which warrants further research and supportive strategies of a structural manner.

## Theoretical Approach

Our analysis of engineering ethics education is inspired by Critical Realism, a theoretical approach that strives to develop deeper levels of explanation and understanding (Mc Evoy & Richards, 2006).

An example relevant to engineering ethics refers to accident causation. Pearce and Tombs (1998) draw explicitly on Critical Realism to argue that the analysis of accident causation tends to concentrate on first-order causes, such as immediate production pressures, poor communication or lack of training, and less on the second-order underlying mechanisms that generate them. Explanations about accidents should place their occurrence within “prevailing systems of economic, social and political organisation, dominant value systems and beliefs, and the differential distribution of power” (Tombs, 2007, p. 29), before exploring their causes, which often are social, political or historical (Dien et al., 2004, 2012). According to Tombs (2007), such analysis should consider factors present at distinct levels, ranging from individual agents to the contexts in which they operate, such as the workplace culture or the political environment in which a company is based.

By drawing inspiration from Critical Realism, our approach responds to arguments for analysing education as a complex and multi-layered system (Bybee, 2003; Godfrey, 2009; Lattuca & Stark, 2009; Sterling, 2004). Sterling (2004) uses an iceberg metaphor to point to the structures of paradigm and purpose guiding policy and practice in higher education, which are mostly hidden from view and consequently from debate. Godfrey (2009) also highlights the need for situating findings related to individual beliefs and practices manifest in engineering education within deeper structures. A similar claim in favour of deploying a depth analysis is made by Lattuca and Stark (2009, p. 303), who argue that the higher education curriculum reflects its socio-cultural context. Nevertheless, higher education research has largely neglected the socio-cultural context that shapes the activities of individuals (Ashwin, 2009; Scott, 2005, 2010; Trowler, 2005, 2008).

Our literature survey comprises four levels of analysis (Table 1), whose main features and interrelations are explored. These four levels are (i) the individual level represented by instructors and students, (ii) the institutional level represented by higher education units such as engineering programmes, departments or colleges, (iii) the policy level represented by national accrediting bodies, and (iv) the wider cultural milieu in which engineering education takes place. A multi-level approach allows us to address some of the limitations of research in higher education, which tends to either include only individual agents such as instructors or students (Ashwin, 2008, 2009; Trowler, 2005, 2008), or to focus on the levels of policy and practice without examining the deeper levels of paradigm and purpose guiding them (Sterling, 2004). By adopting an approach focused on distinct analytical levels, our contribution aims to place individuals in their socio-cultural, institutional and policy context and to link some of the findings in engineering ethics education with wider debates about the dominant paradigm for engineering education (Jamison et al., 2014). A key issue that emerges is the need for clarity about the purpose of engineering education and the mission of reform programmes. The ultimate aim is to develop ground for reflection on the structural strategies needed for effecting change in engineering ethics education and to foster a socio-technical orientation of the engineering curriculum *for* ethics.

**Table 1:** Levels of a critical realist inspired literature review of engineering ethics education

Analytical level	Focus
Individual	Beliefs, understanding and attitudes towards engineering ethics education held by instructors and students Practices of teaching and assessing ethics
Institutional	Beliefs, understanding and attitudes towards engineering ethics education expressed on behalf of a programme, department or institution Practices of implementing ethics and measures targeting ethics in engineering programmes, departments or institutions
Policy	Beliefs, understanding and attitudes towards engineering ethics education of representatives of governmental and accrediting bodies or funding agencies Measures and policies targeting ethics adopted by governmental and accrediting bodies or funding agencies^a^
Cultural	Paradigms of engineering education and practice

^a^As mentioned in the limitations of the review, we are not focusing on funding agencies in this article

The literature review (Wilson & Anagnostopoulos, 2021) relied on the core collection of the Web of Science for identifying research about undergraduate engineering ethics education. To retrieve sources that address issues representing the four analytical levels described in Table 1, the following combination of key terms was used to search in the titles and abstract of publications during the period 2000–2020: “ethic*” AND “engineering” AND “education*” OR “course” OR “curricul*” OR “instruct*” OR “teach*” OR “assess*” OR “implement*” OR “challeng*” OR “accredit*” OR “cultur*”.

To ensure a more comprehensive analysis, the process of retrieving sources based on keywords search was followed by an overview of the references mentioned by the most cited publications, for identifying additional publications relevant to the objectives of the analysis that do not have this combination of key terms in their title or abstract. An additional search was then undertaken in the engineering education journals and conference proceedings that featured the highest number of publications during the first search process. More specifically, the first author searched the databases of the Journal of Engineering Education, the European Journal of Engineering Education and Science and Engineering Ethics, as well as the conference websites for the American Society for Engineering Education and the European Society for Engineering Education to retrieve additional publications featuring the word “ethics” in their title, abstract or keywords.

A limitation that emerged during the source retrieval process relates to the extensive research published in English and the overemphasis on research undertaken in the US, UK, Australian and Western European context, to the exclusion of potential relevant studies set in other national and cultural contexts. A second limitation is linked with how accurately the published research on engineering ethics education that guides our analysis reflects the reality of teaching and institutional attitudes and practices. While it is not possible to ensure that the totality of teaching and institutional attitudes and practices is represented by existing research, the studies published can be considered a reliable indicator of the challenges and states of affairs in engineering ethics education. A final limitation is due to narrowing the analysis of policy actors to accrediting bodies, thus omitting other influential actors such as funding agencies or state ministries. We are referring here only to accrediting bodies, being modest about the breadth we can ensure in a journal publication and at the same time mindful of the role played by this policy body in engineering education worldwide. We consider that accreditation is a force shaping engineering education in many and various national contexts, in ways that resonate across geographical borders, while the role of other policy actors might be confined to specific geographical contexts.

## Multi-level Analysis of the Challenges of Engineering Ethics Education

In what follows, we present the empirical and theoretical findings about the challenges and dissatisfaction with engineering ethics education reported in the literature, manifest at each analytical level.

### Individual Level

The main challenges experienced by instructors teaching ethics can be subsumed under seven main themes, related to (i) the lack of clarity about the appropriate pedagogical approaches for supporting the various goals set for engineering ethics education, (ii) ensuring a broad coverage of topics, (iii) conducting assessment, (iv) the limited empirical research guiding the design and use of teaching materials, (v) the lack of familiarity with the subject, (vi) the lack of support, and (vii) students’ resistance to ethics.

#### Diversity and Lack of Clarity for Goals Set in Engineering Ethics Education

The limited research on the effectiveness of the various strategies and goals set for engineering ethics education is a major challenge revealed in the literature. According to Hess and Fore (2018), there are multiple ethics related learning goals, and no consensus on which strategies are the most effective towards these goals, nor goals should be prioritised. The instructors surveyed by Romkey (2015, p. 25) were found to employ a “very diverse” set of overall teaching goals, but “the goals and practices did not always align”. As stressed by Keefer et al., (2014, p. 250), “variability in instructional goals within the same content areas raises the spectre of significant problems with curricular alignment”. A coherent strategy implies that the goals set for engineering ethics education inform decisions about assessment (Borrego & Cutler, 2010, p. 366), and are congruent with the delivery and pedagogical methods employed (Li & Fu, 2012, p. 343). The lack of clarity and alignment might lead to missed educational opportunities (Li & Fu, 2012).

The goals proposed for engineering ethics education can be grouped under 12 major categories, as seen in Table 2. Inspired by the goals described by Van de Poel and Royakkers, (2011), six of these categories relate to the development of moral sensibility, analysis, creativity, judgement, decision-making and argumentation. Additionally, we identified goals that fall under categories such as moral knowledge, design and agency, situatedness, emotional and character and virtue development.

**Table 2:** Goals posited for engineering ethics education

Categories	Goals
Moral sensibility*	Developing proficiency in recognizing social and ethical issues in engineering (Harris et al., 1996; Pritchard, 2005; Van de Poel & Royakkers, 2011; Martin & Schinzinger, 2013) Encouraging students to take ethics seriously (Harris et al., 1996) Increasing students’ sensitivity to ethical issues (Davis, 1999; Harris et al., 1996)
Moral analysis*	Analyzing moral problems in terms of facts, values, stakeholders and their interests (Van de Poel & Royakkers, 2011) Comprehending, clarifying, and assessing arguments on opposing sides of moral issues (Martin & Schinzinger, 2013) Facilitating the analysis of key ethical principles (Harris et al., 1996) Exploring the perspective of those in other positions **(**Lynch & Kline, 2000; Martin et al., 2019)
Moral creativity*	Considering different options for action in the light of (conflicting) moral values and relevant facts (Van de Poel & Royakkers, 2011) Stimulating ethical imagination (Coeckelbergh, 2006; Harris et al., 1996; Martin & Schinzinger, 2013; Pritchard, 2005) Creatively exploring solutions rather than choosing a dilemma horn (Lynch & Kline, 2000) Enhancing divergent thinking (Haws, 2001)
Moral judgment*	Making moral judgments based on different ethical theories or frameworks, including professional ethics and common-sense morality (Van de Poel & Royakkers, 2011) Improving ethical judgement (Davis, 1999; Harris et al., 1996; Pritchard, 2005), understood as the ability to reliably respond to any situation with a course of action that makes life better (Davis, 2012) Forming consistent and comprehensive viewpoints based on consideration of relevant facts (Martin & Schinzinger, 2013)
Moral decision-making*	Enabling students to make decisions based on different ethical theories and frameworks (Van de Poel & Royakkers, 2011) Providing conceptual tools for reflecting on how organizational practices can potentially threaten public safety and welfare and how to counter the normalization of deviance (Lynch & Kline, 2000) Helping students deal with ambiguity in decision-making situations (Harris et al., 1996)
Moral argumentation*	Developing the ability to morally justify one’s actions and to discuss and evaluate them (Van de Poel & Royakkers, 2011)
Moral knowledge	Gaining knowledge of professional standards, codes and principles (Davis, 1999; Harris et al., 1996; Pritchard, 2005) Giving students access to the language of ethics to express and support one’s moral views adequately to others (Haws, 2001; Martin & Schinzinger, 2013) Grounding one’s views and decisions in moral theory (Lynch & Kline, 2000)
Moral design	Considering how values, as well as modes of use and interaction, can be implicitly or explicitly inscribed into engineering artefacts at the design stage (van de Poel & Verbeek, 2006; Verbeek, 2008)
Moral agency and action	Responding wisely and responsibly to situations in a way that satisfies as many potentially Competing constraints as possible (Whitbeck, 1995) Empowering students to reshape the social, economic and legal context of practice (Conlon & Zandvoort, 2011) Encouraging students to take an activist stance “for what is right, good and just” (Hodson, 1999) Inspire the engineers of the future to challenge the status quo and to strengthen the profession (Lawlor, 2021)
Moral character and virtuous development	Increasing students’ ethical willpower (Davis, 1999; Harris et al., 1996) Cultivating students’ sense of professional identity (Loui, 2005; Miller, 2018) Cultivating virtues, such as respect for nature for engaging in environment-friendly engineering (Harris et al., 2019), *phronesis* for identifying certain decision situations and actions as ethically relevant (Frigo et al., 2021), objectivity, care and honesty (Moriarty, 2009; Nair & Bulleit, 2020)
Moral emotional development	Reflecting on the role of emotions in the development and acceptability of risky technologies (Roeser, 2012) or in the effects of climate change (Lönngren et al., 2020) Engaging learners in their emotional life as to develop a sense of empathy with people across physical, social and cultural distances and a language for emotions (Tormey, 2005; Hess & Fila, 2016; Hess et al., 2017)
Moral situatedness	Understanding the social relations of expertise in connection with technology management and decision-making (Devon, 1999) Helping students situate their work in its contribution to their community (Haws, 2001) Acknowledging the social dimension of engineering practice (Martin et al., 2019)

*Category borrowed from Vande Poel and Royakkers (2011)

There is limited research exploring the prevalence of each learning goal in engineering ethics instruction or on the teaching methods and content to achieve them, which raises questions on how to ensure curricular alignment. Furthermore, there is little known on how specific learning goals might convey to students an understanding of the societal mission of engineering, as captured by the broader theoretical frameworks used to conceptualise engineering ethics education.

Considering the more popular theoretical frameworks developed in the last decades, learning goals can be further subsumed under microethics, macroethics, virtue ethics, value sensitive design and feminist ethics of technologies.

The microethical model is characterised by a strong emphasis on the individual responsibility of engineers (Herkert, 2005). Basart and Serra (2013, p. 179) capture the spirit of microethics by noting that it “is usually focused on engineers’ ethics, engineers acting as individuals.” It strives to expose students to ethical dilemmas, with goals focused on enhancing students’ professional responsibility through knowledge of professional codes and refining their moral judgement. This is the theoretical approach considered to prevail in engineering ethics education (Bielefeldt et al., 2016; Colby & Sullivan, 2008; Herkert, 2000; Hess & Fore, 2018).

The macroethics model moves beyond an understanding of engineering actions and responsibilities in individual terms towards engaging the engineering profession as a whole and reflecting on the profession’s responsibility in technological development (Vanderburg, 1989; Herkert, 2005). The focus is on the collective responsibilities of engineers and societal decision-making about technology (Herkert, 2005, p. 373). Goals address the context of engineering practice in order to enable an engineer’s agency to act ethically (Zandvoort et al*.*, 2008; Conlon, 2011; Chance et al., 2021). Macroethical goals also target the development of technologies that are congruent with egalitarian and democratic structures and institutions (Vanderburg, 1989), or foster the active involvement in public policy to formulate rules and regulations promoting socially just practices (Martin & Schinzinger, 2013, p. 29; Conlon & Zandvoort, 2011).

Representative of virtue ethics approaches are goals that emphasise the importance of context sensitivity and the acquisition of moral virtues and practical judgment (*phronesis*) for dealing with concrete situations (Nair & Bulleit, 2020). The focus of virtue ethics lies not on the rightness of engineering decisions, actions or outcomes, but on developing the moral attitudes or virtues of the deciding agents that would incline an engineer’s actions (Hillerbrand & Roeser, 2016; Schmidt, 2014; Vallor, 2016). According to virtue ethics, pedagogical approaches that focus on moral action and its consequences need to be complemented by training the future engineer to develop certain character traits or virtues. Virtue ethics has been posited as a more appropriate frame to convey aspects of engineering professionalism, such as sensitivity to risk, awareness of the social context of technology, respect for nature and commitment to the public good (Harris, 2008). Virtue-based pedagogical approaches are also considered to improve engineering students' ethical competence, contributing to learning goals purporting to an enhanced ethical sensitivity, awareness, analysis and judgement (Frigo et al, 2021). This theoretical approach lies at the basis of Bowen’s (2009) understanding of the mission of engineering as enhancing the quality of human life, the well-being of the community or the vitality of the eco-system. Fostering a virtue based approach in engineering education can contribute to the development of students’ professional identity as “virtuous engineers”, who can

assert their responsibility for engaging in a combined human performance that involves the exercise of practical judgment to enhance the material well-being of all people by achieving safety, sustainability and efficiency while exhibiting objectivity, care and honesty in assessing, managing and communicating risk. (Schmidt, 2014, p. 1007)

An alternative theoretical approach which aims to integrate micro and macro ethical aspects in engineering education is Value Sensitive Design.^2^ Introduced by Friedman (1996) and later popularised in the Netherlands, VSD draws on the philosophy of technology and Science and Technology Studies to connect the moral analysis of the influence exercised by technological artefacts on their environment with moral decision-making during the design process (van de Poel & Verbeek, 2006; Verbeek, 2008, 2011). A major goal of this approach is to make students aware of how the effects of a technological artefact transcend its functionality. When technologies fulfil their functions, they also shape the experiences and actions of their users (Verbeek, 2006). VSD thus proposes a broadening of the scope of engineering ethics education as to encompass goals fostering the professional responsibility of engineers from the design stage of an artefact, by considering the prospective mediating role of technology development and instilling it with moral values (Verbeek, 2008, 2011). The values prioritised by this approach target the societal good over instrumental values aimed at enhancing economic profit (Friedman et al., 2013). Important values promoted by VSD relate to safety, sustainability and inequality (Mok & Hyysalo, 2018; Mouter et al., 2018; van Gorp, 2005). The focus is on encouraging students to design value driven artefacts and solutions that contribute to societal welfare or diminish the negative societal effects of existing technologies (Gorman, 2000, 2001; van Gorp & van de Poel, 2001; Verbeek, 2008, 2011).

A feminist philosophy of technology is an inclusive and value-laden approach that employs a critical discourse on modern technological development (Loh, 2019). In the articulation of feminist philosophy of technology, the concern lies with the development of tools and knowledge for enhancing women’s “ability to develop, expand, and express their capacities” (Layne, 2010, p. 3). The goals of this approach range from addressing the status of women to restructuring social arrangements in ways that adjust the power relations between genders (Layne, 2010). These goals are aligned with the precepts of VSD (Pantazidou & Nair, 1999; Whitbeck, 1998), by reflecting on the gendered assumptions inherent in technological design and promoting the development of technological artefacts that do not discriminate against the female gender (Michefelder et al*.*, 2017; Riley, 2013). Thus, for feminist philosophy of technology, technological artefacts cannot be divorced from the social, political and economic context of their development and modes of use (Layne, 2010; Whitbeck, 1998). In this sense, feminist philosophy of technology has a common history and agenda with social justice movements, through the focus on ending “different kinds of oppression, to create economic equality, to uphold human rights and dignity, and to restore right relationships among all people” (Riley, 2008, p. 5; Riley et al., 2009).

Mindful of the varied theoretical frameworks for organizing the goals of engineering ethics education, we suggest that curricular alignment should consider not only the teaching and assessment methods or the thematic content of instruction, but also the view of the mission of engineering put forward by different conceptualisations. The prevailing goals reported in the literature have an overriding focus on the moral agency of engineers and less on the context in which they may have to make ethical decisions or on the values embedded at the design stage (Hess & Fore, 2018). This means that students might be exposed to a singular and narrow dimension of engineering ethics (Canney et al., 2017). To counteract this risk, microethical approaches can be complemented by other theoretical approaches, as to ensure the attainment of a broader spectrum of ethics learning goals and a nuanced view of engineers’ role in society, reflective also of the institution’s educational vision and graduate attributes.

Furthermore, there are also concerns that ethics education might lead to indoctrination. Some instructors argue against the presence of ethics in the engineering curriculum, considering it a subjective and personal issue falling under the responsibility of the students’ families (Romkey, 2015; Vesilind, 1991; Walczak et al., 2010). This stance highlights the need for open discussions and clarifications on the object of engineering ethics, as to explore and challenge common intuitions and how the personal understanding of the subject is reflected in the aspirational goals set for ethics.

#### Content of Engineering Ethics Education

Engineering ethics is taught using diverse content areas. The major content areas identified include responsibility, sustainability, health and safety, legislation, professional ethics, community engagement and humanitarian engineering, societal context, value sensitive design, academic and research integrity, ethical theories, business studies and military applications (Bielefeldt et al., 2019a, 2019b; Haws, 2001; Kline, 2001; Lynch, 1997; Martin et al., 2020, p. 2). At the core of engineering ethics lies the concept of "professional responsibility” (Herkert, 2002), understood by Whitbeck (1998) as the “exercise of judgment and care to achieve or maintain a desirable state of affairs”.

However, not all content areas are of “equal value” for the goal of helping engineers connect their work to the broader community and exercise their societal responsibility (Haws, 2001, p. 227). More so, there is an uneven coverage of key ethical issues (Colby & Sullivan, 2008; Polmear et al, 2019), which is consistent with the difference in how instructors and students perceive the coverage of ethics. Even though faculty describe their instruction as including not only codes, but also a nuanced treatment of complex issues, students report hearing “simplistic, black-and-white messages about ethics” (Holsapple et al., 2012, p. 101). This might be due to the instructors’ lack of familiarity and training in teaching ethics, such that simplistic teaching might lead to simplistic messages.^3^

Reflecting on the uneven coverage of engineering ethics education, Bielefeldt et al. (2016) note that there is a limited understanding of the extent to which macroethical topics are being addressed. While the focus is on professional codes, safety and plagiarism (Atesh et al., 2017; Colby & Sullivan, 2008, pp. 329–330; Hess & Fore, 2018, p. 551; Mitcham, 2017, p. 4; Polmear et al., 2018, p. 14), there are concerns that macro topics have lesser prominence. Under-emphasized topics include equity, the critical histories of ideas about engineering, the broader mission and implications of the profession, as well as the respect for life, law and public good (Atesh et al., 2017; Colby & Sullivan, 2008; Mitcham, 2009; Rottman & Reeve, 2020). According to Mitcham (2009), discussions about public safety, health and welfare should be complemented by reflection on their historical and social character.

#### Conducting Assessment

As Goldin et al., (2015, p. 790) point out, the instructors’ teaching approach affects assessment, and “given the variations in teaching applied ethics, one must be clear about the goals of teaching, and the real opportunities for assessment.” Keefer et al., (2014, p. 259) also highlight the importance of aligning goals with teaching methods as to ensure they are “appropriately assessed”, noting that alignment is “still a weakness in the present state of ethics education.” The assessment of ethics raises several challenges, pertaining to the unfamiliarity with evaluating and grading the ethical components of engineering courses, as well as to the limited guidance about what assessment methods are suitable for nontechnical subjects (Goldin et al., 2006; Romkey, 2015; Sinha et al., 2007).

Engineering ethics instructors typically use between 0 and 4 assessment methods, with an average of two assessment methods per course (Bielefeldt et al., 2016, p. 12). Popular assessment methods include reflective essays and individual assignments graded with a rubric (Bielefeldt et al., 2016, p. 12), as well as presentations, group projects and portfolios (Sunderland et al., 2013). Nevertheless, it is more common for ethical components either to remain unassessed or be subjected to a binary assessment as pass/fail (Keefer et al., 2014, p. 251), with several instructors indicating they “made no effort to assess student’s understanding of ethics” (Freyne & Hale, 2009, p. 8).

According to Newberry (2004, pp. 349–350), the use of varied assessment methods is linked to a personal understanding of engineering ethics by instructors unfamiliar with this subject. Davis and Feinerman (2012) also highlight the difficulty in grading students on ethical abilities and character. Many of the faculty with a technical background consider ethics to be a personal and subjective subject, ignoring how Humanities faculty assess students’ work and provide feedback (Davis & Feinerman, 2012). The assessment of case study assignments can also be challenging due to the ill-structured nature of the problems they address (Goldin et al., 2015).

These challenges led to a call for the development of standardized assessment instruments, scoring rubrics and instruments. There are currently instruments that measure the maturity of students’ reflection on ethical issues (Rest, 1979; Rest et al., 1999), the influence of formal and informal ethical experiences on students’ behaviour (Finelli et al., 2012; Harding et al*.*, 2013), students’ views on social responsibility (Canney & Bielefeldt, 2016), their moral sensitivity (Borenstein et al., 2008), the ability to address ethical dilemmas, focused on attributes of attainment such as the recognition, argumentation, analysis, perspective taking and resolution (Sindelar et al., 2003), moral reasoning (Borenstein et al., 2010), moral decision-making in design projects (Zhu et al., 2014) or in the context of briefs provided by industry stakeholders (Moskal et al., 2001) and real world scenarios (Bagdasarov et al., 2016; Mumford et al, 2006).

An advantage of assessment instruments is that results can serve as feedback for instructors in the process of curricular improvement, revealing where to allocate future instructional resources (Keefer et al., 2014, p. 258; Moskal et al., 2001; Sindelar et al., 2003). A significant drawback is that none of the instructors surveyed by Bielefeldt et al. (2016) has been using a standardised assessment method, as they are unaware of their existence. This might be linked to a lack of familiarity with ethics and training in ethics instruction. Further drawbacks refer to the lengthy time duration of standardised assessment tests or their lack of relevance across different student cohorts (Davis & Feinerman, 2012). As Davis and Feinerman (2012, p. 357) note, standardized assessment offers “no middle ground for a test both general enough to produce comparable results across a wide range of courses and specific enough to measure what was actually learned in a particular course”.

More so, the quantitative treatment of ethical matters put forward by standardised tests can be interpreted as an attempt to bring the positivist approach characteristic of the technical culture into a nontechnical subject. Also notable is the Western centric nature of existing standardized tests. The aforementioned tests have been developed in the US and might exclude the cultural traditions of other geographical regions or the individual characteristics of respondents that are shaped by their gender, ethnicity, cultural background or social class (Zhu et al., 2014, p. 10). Goals associated with the feminist and value-based design approaches are also missing from the scope of existing standardized tests.

#### Lack of Expertise

As Barry and Herkert (2014, p. 824) note, the preparation of faculty to “comfortably engage” with the subject remains one of the biggest challenges facing engineering ethics education. Other major challenges encountered by instructors relate to formulating ethical learning goals and understanding the expectations of accrediting bodies as to how these can be achieved (Besterfield-Sacre et al., 2000; Colby & Sullivan, 2008; Herkert, 2002; Sheppard et al., 2009). At the root of these challenges, we find the instructors’ lesser familiarity with ethics, which makes difficult finding appropriate pedagogical content and linking ethical concerns with technical subjects (Barry & Herkert, 2014). Furthermore, engineering instructors highlight the lack of guidance and training on how to teach ethics (Harding et al., 2009; Monteiro, 2016; Polmear et al., 2018; Romkey, 2015; Sinha et al., 2007; Vesilind, 1991; Walczak et al., 2010). Also notable is the time commitment required for becoming acquainted with an unfamiliar subject, which is an impediment given the busy schedule of faculty members (Walczak et al., 2010).

Co-teaching activities involving engineering and philosophy or social sciences instructors can address the problem of expertise and convey to students a message about the importance of this subject. Nevertheless, it is an expensive, time and labour-intensive approach, which requires long-term contact and research efforts (Bombaerts et al., 2021). Moreover, this approach is considered “second-rate academic work” (Taebi & Kastenberg, 2019, p. 1768), and is not properly acknowledged in promotion and hiring schemes (National Academy of Engineering, 2017, p. 12).

#### Empirical Research Guiding the Design and Use of Teaching Materials

Existing studies report the use of various teaching methods (Harding et al., 2013; Keefer et al., 2014). These include case studies, lectures and presentations, role-playing activities, in-class or online discussion, debates, voting, games, online courses, films and videos, creative fiction, science fiction, community service, field trips and visits (Loui, 2009; Alpay, 2011; Atwood & Read-Daily, 2015; Berne & Schummer, 2005; Bielefeldt et al*.*, 2016; Burton et al., 2018; Finelli et al., 2012; Génova & González, 2015; Itani, 2013; Kang & Lundeberg, 2010; Lloyd & van de Poel, 2008; Loui, 2000; Lumgair, 2018; Pritchard, 2000; Rabb et al., 2015; Voss, 2013). One of the most popular methods for teaching engineering ethics are case studies (Colby & Sullivan, 2008; Herkert, 2000; Yadav & Barry, 2009).

Nevertheless, despite the variety of teaching methods and the prevalence of case studies, there is limited empirical research that could elucidate the effectiveness of each teaching approach towards the attainment of clearly defined goals, as well as their impact on student engagement (Bagdasarov et al., 2013; Bombaerts et al., 2021; Martin et al., 2021; Thiel et al., 2013, p. 267). There is also little known on how cases are presented in engineering ethics instruction and the kind of cases used (Yadav et al, 2007), how they should be taught (Davis & Yadav, 2014, p. 172), and what approach serves the achievement of which learning goals (Romkey, 2015). As such, one cannot point to the approach by which the case method could achieve its “alleged superiority” in engineering ethics instruction (Abaté, 2011, p. 589).

#### Lack of Support

Another challenge faced at individual level is the lack of peer and institutional support for instructors teaching ethics (Polmear et al., 2018; Romkey, 2015; Walczak et al., 2010). Engineering ethics instructors report feeling isolated and lacking a peer group within their institution with whom they could discuss their teaching approaches (National Academy of Engineering, 2017). Recent initiatives for connecting engineering ethics instructors and researchers include the Engineering Ethics Division of the American Society for Engineering Education, the special interest group on ethics of the European Society of Engineering Education, or the Communities of Practice supported by the Online Ethics Center for Engineering and Science.^4^ Instructors also report resistance encountered at the institutional level, related to fewer resources allocated for ethics teaching and promotion systems that do not recognise the value of ethics education (Martin et al., 2021; Polmear et al., 2018, p. 13; Taebi & Kastenberg, 2019; Walczak et al., 2010).

#### Student Reception

Students’ skills and reception of ethics is another major challenge of engineering ethics instruction (Harding et al., 2009; Romkey, 2015). Students tend to show disinterest, resistance, and difficulties when exposed to ethics and societal considerations (Bairaktarova, & Evangelou, 2011; Polmear et al., 2018, p. 9), as well as a lack of emotional engagement with the course content (Balakrishnan & Tarlocha, 2015; Newberry, 2004). Students also prefer to have ethics as a non-compulsory topic that is not assessed (Sucala, 2019), and invest less time preparing for ethics courses (Bombaerts & Nickel, 2017; Martin, 2020).

This may contribute to a trend identified in several research studies showing that students’ engagement with public welfare and their moral reasoning decreases throughout their engineering studies (Bielefeldt & Canney, 2016; Cech, 2014; Rulifson & Bielefeldt, 2018), even upon receiving ethics training (Tormey et al., 2015). Cech (2014) found that students from engineering programmes which emphasise the development of technical skills to the detriment of ethics and social engagement tend to have declining beliefs about the importance of public welfare from their first to last year of studies, and their engagement with public welfare issues does not rebound upon entering the workplace. Engineering students tend to develop strong and rigid views about the lower value of academic subjects oriented towards people and society (Adams et al*.*, 2018). They also express less commitment to social activism and concern for society than students from other disciplines (Sax, 2000), and consider unrealistic to expect engineers to have an ethical behaviour (Stappenbelt, 2013).

### Institutional Level

Barry and Herkert (2014, p. 420) highlight that the key aspects in the implementation of ethics at programme level refer to where and how ethics is integrated in the programme and the weight given to ethics. These questions touch on issues considered challenging at the institutional level, such as what constitutes an effective design and implementation of ethics in the engineering curricula, as well as ensuring the balance between technical and ethical content (Sheppard et al., 2009; Wicklein, 1997).

#### Low Emphasis on Ethics

As Wicklein (1997, p. 74) remarks, it is important to enquire to what degree should the engineering curriculum be devoted to technical skill training, given that historically there has been “an exorbitant amount of instructional time to this area, while slighting many of the other facets of the curriculum”. According to Wicklein (1997, p. 74), the key to a healthy engineering curriculum is finding the “appropriate balance of tool skills with other curricular areas”.

Empirical research paints an educational landscape where ethics has marginal presence in the engineering curriculum, even in educational systems where ethics features among the accreditation criteria (Barry & Ohland, 2012; Colby & Sullivan, 2008; Ocone, 2013). The self-assessment conducted by engineering programmes for the purpose of accreditation in Ireland reveals that ethics is the accreditation outcome with the lowest weight in the engineering curriculum, compared with both technical and nontechnical outcomes (Martin, 2020). In countries where ethics education is not mandatory for accreditation, ethics is mostly absent (Monteiro et al., 2016, 2017). As Mitcham (2014) points out, humanities and social science requirements are often limited to “little more than a semester’s worth, spread over a degree program crammed with science and engineering”. The marginal role of ethics in a technically dominant curriculum is revealed also by the few number of exams and assignments addressing ethical considerations (Fabregat, 2013; Miñano et al., 2017; Stonyer, 1998).

There is a disparity between the perceived importance of ethical and societal related practices and their presence in the curriculum (Romkey, 2015, p. 14). The main risk associated with a weak presence of ethics in engineering education is that of conveying to students the message that ethics is not as important for their education and future profession as the development of technical abilities (McGinn, 2003, p. 525). Given that university education is the propitious period when engineering students start developing their identities as future professionals (Loui, 2005), the curricular weight given to ethics is of crucial importance for sending students the message that ethics is not peripheral to engineering, but a substantial aspect of their profession (Li & Fu, 2012; Trevelyan, 2010, 2014).

#### Lack of a Systematic Approach Driving the Implementation of Ethics

For implementing ethics in a systematic manner, a cohesive and purposeful strategy needs to be designed at institutional level. To ensure a cohesive curriculum, devising an implementation strategy should take precedence over the introduction of ethics learning activities (Li & Fu, 2012). Such strategy should be considerate of quality assurance mechanisms, accreditation requirements, and strive to adapt the implementation of ethics as to fit the vision and graduate attributes set by the institution as well as the specific characteristics of the institution’s ecosystem.

A systematic implementation of ethics requires a wide scale transformation undertaken at institutional level (LeBlanc, 2002). The challenges of such an endeavour are rooted in budgetary pressures, limited institutional resources for bringing external instructors with an expertise in this area, insufficient space in the curriculum and lack of guidance (Romkey, 2015; Sheppard et al., 2009; Walczak et al., 2010).

Besterfield-Sacre et al., (2000, p. 100) note that when a dedicated ethics criterion was introduced in the US, there was “much concern as to how to best operationalise each outcome for use within one’s own institution.” A similar deficit about the operationalising the accreditation outcome dedicated to ethics in the engineering curriculum is encountered in the context of engineering education in Ireland, where Murphy et al., (2019, p. 381) found no evidence that any institution implemented ethics “to set itself apart […] as different and unique” and there are “no clear themes reflecting an institute-wide focus” with respect to ethics.

According to Herkert (2002), the vagueness of the accreditation criterion “makes it difficult to implement a standard model for teaching engineering ethics.” A significant challenge is thus linked to understanding the formulation of accreditation requirements purporting to ethics and the expectations of the accreditation body about the implementation of ethics (Colby & Sullivan, 2008; Sheppard et al., 2009). Furthermore, engineering programmes report the lack of “consistent, accurate, and reliable methods of teaching ethics and measuring its outcome” (Bairaktarova & Woodcock, 2015), pointing to issues related to quality assurance. This is reflected in the disparity of approaches for teaching and assessing ethics (Bielefeldt, 2016; Harding et al., 2013), and the call for a constructive alignment between programme outcomes targeting ethics, assessment methods and the design of learning environments (Bombaerts et al., 2019; Borrego & Cutler, 2010).

The unconstructive feedback following accreditation events and the lack of guidance from the accrediting body on how to operationalise the outcome has been highlighted as a significant barrier in the systematic implementation of ethics at institutional level (Barry & Ohland, 2012; Bielefeldt et al., 2016; Herkert, 2002; Murphy et al., 2019, pp. 381–382). According to Barry and Ohland (2012, p. 389), the feedback on ethics received from the accrediting body is “either significantly lacking or not constructively useful to the evaluated programmes,” which might impede the dimension of the accreditation process associated with quality assurance and improvement (Kumar et al., 2020; Quiles-Ramos et al., 2017). Barry and Ohland (2012, p. 389) further stress that the lack of feedback following the accreditation review has left “most programs uncertain of their chosen quantity of curricular content". Reflecting on the South African context, Gwynne-Evans et al., (2021, p. 10) note that the description of the graduate attributes set by the Engineering Council of South Africa provides “very little conceptual detail” as to their meaning, which results in “insufficient signposts to guide educators in the implementation and assessment of ethics within the engineering programme”.

The vagueness and limited scope of the ethics accreditation criterion risks leading to a narrow treatment of the subject (Gwynne-Evans et al., 2021; Riley, 2021). Bielefeldt et al. (2016) are especially concerned that in the US, ABET’s self-study documents do not distinguish between micro and macro ethical issues, while the common use of the Fundamentals of Engineering exam implies a focus on microethical issues.

There also appears to be a less thorough evaluation of how engineering programmes meet the accreditation criterion dedicated to ethics, compounded by minimal recommendations on the implementation of this outcome, and a granting of accreditation irrespective of the lacuna identified in the evidence purporting to ethics. Such absence can lead to minimal interventions undertaken at programme level targeting ethics. Examining the Irish context, Murphy et al., (2019, p. 381) found “no evidence of systemic attention to a broadening agenda within the accreditation reports”, and that “often, the same (few) courses” within a programme are mentioned as bearing the responsibility to provide all the evidence for meeting the requirement purporting to ethics. Ethics thus ends up being regarded as an “add-on” implanted artificially in an engineering programme, rather than implemented following a programme wide strategic process (Flynn & Barry, 2010; Martin, 2020; Murphy et al., 2019; Newberry, 2004; Polmear et al., 2018; Sunderland, 2019).

A survey of 100 programmes offered by 40 engineering schools in the US, found that few schools managed to institute “systematic programmes to educate for a broad sense of professional responsibility” (Colby & Sullivan, 2008, p. 330). In Ireland, a similar ad-hoc implementation of ethics has been reported, contrasted with the carefully designed strategy driving the implementation of technical topics. According to an evaluator for the accrediting body,

“if you take technical subjects, like structures or signal processing, the academics will make sure that the design of the programme incorporates these, and in a logical and coherent way. But they do not take the same approach about the ethical material” (Martin, 2020).

The challenges of implementing ethics are compounded by questions of how to make room for new content in a crowded curriculum. Technical and scientific subjects are given priority in the engineering curriculum, making it difficult for programmes to decide which technical components should be reduced to introduce new ethical components (Harding et al., 2009; Polmear et al., 2018; Romkey, 2015; Walczak et al., 2010).

### Policy Level

At policy level, the impact of national accrediting bodies on the engineering curriculum was highlighted as a potential force for an enhanced role given to ethics. Since the adoption of the Washington Accord, signatory countries are required to align to a similar set of graduate attributes, including ethics, and their accrediting bodies ensure that these are being met. As such, the introduction of an accreditation criterion dedicated to ethics in the Washington Accord signatory countries has led to an increase in the number of courses addressing ethical issues (Barry & Ohland, 2012; Lattuca et al., 2006; Martin, 2020; Ocone, 2013; Skinner et al., 2007; Volkwein, et al., 2004).

Despite the positive influence of accrediting bodies on enhancing the presence of ethics in the engineering curriculum through the formulation of required outcomes, there are doubts that the pressure from accreditation criteria can inform deeper curricular change (Little, 2019; Sunderland, 2013).

#### Role of a Dedicated Accreditation Criterion

Having an accreditation criterion dedicated to ethics can contribute to its increased presence in the engineering curriculum. In the US, the formulation of the accreditation criteria known as EC2000 constituted a step forward towards the inclusion of more societal and environmental topics, as well as of considerations regarding the professional and ethical responsibilities of engineers (Herkert, 2001; Johnston & Eager, 2001). Prior to the adoption of EC2000, the engineering academic landscape in the US was described as neglecting the ethical dimension of the profession (Herkert, 2002, 2005). A survey of US course catalogues conducted by Stephan (1999, pp. 460–461) showed that in 1998, less than 27% of colleges had a mandatory course addressing ethics. Later studies have indeed confirmed an increase in the number of mandatory ethical courses, provided either by engineering programmes or by humanities programmes within the same institution (Barry & Ohland, 2012; Volkwein et al., 2004). Furthermore, a study commissioned by ABET noted an “increased emphasis on nearly all of the professional skills and knowledge sets” associated with the accreditation criterion dedicated to ethics (Lattuca et al., 2006, p. 3). Surveys covering the period prior to the introduction of the EC2000 showed that undergraduate engineering students did not perceive the importance of learning about the engineer’s role in society (Peters, 1998, p. 874), considering that their courses prepared them “only a little bit or not at all” to face ethical issues in the workplace (McGinn, 2003). In contrast to their counterparts who graduated prior to the introduction of EC2000, 2004 graduates reported higher ability levels on outcomes related to the awareness of the impact of engineering decision-making and ethics (Lattuca et al., 2006, p. 9).

While empirical research on the reception and impact of an accreditation criterion dedicated to ethics is predominantly US based, research conducted in Australia and the UK reveals similar findings about the increased curricular presence of ethics following the introduction of such requirements. In Australia, the accreditation criteria were redesigned in 1997 to include the “understanding of the social, cultural, global and environmental responsibilities of the professional engineer, and the need for sustainable development” and an “understanding of the principles of sustainable design and development” (Institution of Engineers, Australia, 1997). A survey of Australian engineering institutions prior to the introduction of a dedicated ethics criterion showed that “apart from a few mentions of sustainability and professionalism, there was no indication of any scholarly interest in these areas” (Johnston et al., 2000, p. 317). Afterwards, the accreditation of engineering programmes required an “integrated exposure to professional engineering practice, including management and professional ethics in not less than 10% of courses, up to a coverage of 20%” (Skinner et al., 2007, p. 136). In the UK, a survey supported by the Royal Academy of Engineering revealed that engineering ethics instruction was “rather patchy” prior to the introduction of ethical specifications in accreditation (Ocone, 2013, p. 263). In Ireland, instructors also perceive an increase in the content dedicated to ethics following the introduction of a dedicated accreditation criterion, from “virtually nothing” (Martin, 2020).

At the same time, the lack of a firm stance of the accrediting body on ethics was found to negatively affect the presence of ethics in the engineering curriculum, as in the case of Portugal (Monteiro & Leite, 2021; Monteiro et al., 2016, 2017, 2019). Monteiro (2016, p. 2) explains the low emphasis given to ethics as the outcome of the strong influence of instructors on shaping curriculum development, based on their own views of education and knowledge. Such views have a cultural root, purporting to the technically oriented education that engineering instructors received (Monteiro, 2016).

#### Surface Level Change

Accreditation requirements can offer the impetus for curricular redesign (Graham, 2012; Lattuca & Stark, 2009; Lewis, 2016). Nevertheless, institutional change driven solely by the demands set by accrediting bodies leads to a culture of compliance rather than of transformative change (Little, 2019). The pressure originating in the interplay between the external influence of accrediting bodies and administrative leadership is considered to marginalise the role of individual instructors (Suskie, 2015), giving rise to a “transactional environment” (Little, 2019, p. 33). As such, the implementation of accreditation recommendations is not considered to necessarily translate into quality curricular change (Bolden, 2007; Haviland, 2014; Kuh et al., 2015).

More so, older universities with a long legacy of alumni are also more resistant to changing their curricula for the purpose of accreditation. Klassen (2018) found that institutional prestige can be used to resist a perceived misinterpretation of criteria by accreditors, in ways that would not be possible in lower status institutions. Elite universities can thus maintain their position with “less need to change their discourse or organisation to maintain their power and position” (Bernstein, 2000, p. 69).

Although policy agents have the role of initiating change through the formulation of mandatory graduate learning outcomes, this effort can nevertheless fall short of achieving a deeper change in the ethos of engineering programmes and of prompting reflection on the purpose of engineering education. Even in national systems of engineering education that have mandated ethics, “one can take a ‘tick box’ approach to the teaching of ethical issues” (Flynn & Barry, 2010, p. 2). As Sunderland (2013, p. 1771) points out, while ethics is meant to be a central component of the contemporary engineering curriculum, it is often perceived as “a marginal requirement to be fulfilled.”

### Cultural Level

Having examined the practices and beliefs manifest at the individual, institutional and policy levels of engineering education, the attention is now moved to the structural forces related to the culture of engineering and engineering education affecting them. First, we establish the legitimacy of the concept of engineering culture, before exploring how the culture of engineering education is understood and its implications for identity development.

#### Engineering Disciplinary Culture

We are guided in the use of the concept of “culture” by the definition provided by Schein (1992, p. 12) and popularised in engineering education research by Godfrey and Parker (2010), according to which culture is understood as

a pattern of shared basic assumptions that the group learned as it solved its problems of external adaptation and internal integration, that has worked well enough to be considered valid, and therefore, to be taught to new members as the correct way to perceive, think, and feel in relation to those problems.

As Godfrey (2009, p. 3) points out, this definition focuses on “the deepest, unconscious level of basic beliefs and assumptions, which underpins the more visible cultural manifestations”.

The characteristics of the scientific culture were first cast by Snow (1959) in opposition to the literary culture. Snow argues that scientists and literary intellectuals exist as distinct “cultures in the anthropological sense […], linked by common habits, common assumptions, and a common way of life”. The distinction made by Snow (1959) between the two cultures overlaps with a 200-year-old hierarchisation of sciences, according to which natural sciences are placed at the top of the hierarchy, and social sciences are found at the bottom (Budd, 1989; Cole, 1983). Despite the diffusion of different hierarchies of sciences, they shared the belief that some fields of research, indicated as “harder”, follow a more rigorous research method and are more reliant on data and theories than other fields, described as “softer”, which are ruled by sociological and psychological factors (Fanelli, 2010).

The distinction between “hard” and “soft” sciences touches on the duality between engineering and natural sciences, on one hand, and humanities and social sciences, on the other. It alludes to a valorisation of the “hard” over the “soft” (Storer, 1967), as well as conveying gendered connotations (Keller, 1985).^5^ “Hard” sciences are considered superior to “soft” sciences (Becher & Trowler, 2001, p. 192^6^; Gardner, 2013), which prompted Cassell (2002, p. 179) to remark that in the use of “a barely disguised (tautological) phallic metaphor, ‘hard’ science is more scientific than ‘soft’.” Referring to the “hard” versus “soft” dichotomy, Biglan (1973) notes that this terminology was meant to capture the level of paradigmatic consensus among the individuals within a specific discipline. According to Biglan (1973, p. 202, 210), there is more consensus in the “hard” disciplines in the adoption of a common framework of content and method, while in “soft” disciplines content and method tend to be idiosyncratic.

This conceptualisation of academic disciplines highlights the isomorphism of the different disciplinary cultures, which transcends fields of specialization, institutional affiliations, or geographical characteristics (Becher, 1981, p. 109; Becher, 1994, pp. 153–155; Becher & Trowler, 2001). The distinctiveness of the engineering disciplinary culture appears to be rooted in common practices and behaviours (Godfrey & Parker, 2010, p. 5), while its homogeneity is linked to the role played by professional and regulatory bodies in determining how disciplinary knowledge practices are translated into curriculum material (Ashwin, 2009). What emerges is the legitimacy of talking about a scientifically-oriented culture specific to engineering (Meiksins, 2007, p. 121), which can be “readily recognised from both inside and out” (Herkert, 2001, p. 410).

#### Contending Paradigms of Engineering Education

The paradigmatic nature of engineering presupposes a high degree of consensus and a tightly structured subject matter, which is considered to affect the instructors’ teaching beliefs and practices (Braxton & Hargens, 1996; Braxton et al., 1998; Jones, 2011). Reflecting on the sociotechnical divide posited by Snow (1959) and Petrina (2003, p. 70) considers that the two cultures are reflected in engineering education. Brint et al.’s (2008) survey confirms the existence of two cultures of undergraduate academic engagement rooted in differences between academic majors. The culture of engagement specific to the humanities and social sciences is characterised by individual assertion and interest in ideas and societal aspects, while in natural sciences and engineering, students engage more in problem-solving courses that target the development of quantitative competencies (Brint et al., 2008, p. 390; Cech, 2014; Godfrey, 2003). There seems to also be two cultures of assessment and grading, with differences reported in instructors’ attitudes towards technical versus nontechnical disciplines (Barnes et al., 2001).

More recently, Jamison et al (2014) proposed a tripartite analysis of the different cultures shaping engineering education and their associated views on engineers’ identity. It comprises the academic paradigm of engineering as applied science, which upholds a technical oriented engineering identity, the market-driven paradigm promoting the identity of engineers as innovators and entrepreneurs, and finally, the integrative paradigm of engineering as public service that fosters the identity of students as social reformers and agents of change. Although the latter paradigm represents “a more balanced or comprehensive approach,” it featured less prominently in the history of engineering education or in programs of educational reform (Jamison et al., 2014, p. 255). As Wicklein (1997, p. 72) remarks, there is little curricular innovation in engineering education, which broadly resembles older vocational models focused on “the technical aspects of selected tools and materials.”

The implications for ethics teaching become apparent. Empirical research on the culture of engineering education (Cech, 2014; Godfrey, 2014; Godfrey & Parker, 2010; Schiff et al., 2021) confirms the valorisation of the technical and the marginalisation of the societal dimension of engineering. Tormey et al., (2015, p. 2) notes that students’ declining moral reasoning is the outcome of the culture within their institution, as courses with ethical content are “swimming against the hidden cultural tide of the programme as a whole”. Kim et al (2018) found a pervasive unreflective disengagement of engineering students rooted in a lack of reflection around the ethical or moral dimensions of a given decision or situation. The culture of disengagement and value neutrality manifest in engineering education is the reflection of a profession-wide phenomenon (Cech, 2014; Riley, 2008), which deems anything outside the technical “to be of lesser value or outside the scope of engineering” (Niles et al., 2020b, p. 498).

It appears then that the culture of engineering education has been articulated in terms of a dominant discourse focused on science (Meiksins, 2007), to the exclusion of alternative discourses of philosophy and ethics, environmental studies, politics or sociology (Johnston et al., 1996, p. 33; Pawley, 2008).

#### Generative Engineering Identity

The value of Jamison et al.’s (2014) tripartite analysis of engineering education is that it allows us to link the different paradigms of engineering education to different conceptions of what it means to be an engineer, thus positing a generative view of engineering identity. By engineering identity is understood who counts as an engineer, what does performing the role of an engineer entail and what are the responsibilities of engineers (Murphy et al., 2015).

Engineering identity is typically portrayed as singular and homogenous, rather than as “many types or manifestations” (Rodriguez et al., 2018, p. 259). As such, engineering identity appears to be largely determined by one’s disciplinary culture (Ashwin, 2009; Becher & Trowler, 2001; Biglan, 1973; Toma, 1997; Umbach, 2007). Engineering education enculturates students into a well-established system of practices, meanings and beliefs, while they learn what it means to be an engineer and what is valued by the discipline (Brint et al., 2008, p. 394). In a similar manner, Meijknecht and van Drongelen (2004, p. 448) compare the monolithic identity of engineers rooted in education to that of professions such as medicine, considering that “university is a place of initiation for the tribe of engineers”. As Stonyer (2002, p. 397) points out, academic enculturation leads to a specific dominant socio-historical engineering identity, as “nuts and bolts” technicists (Faulkner, 2007).

Although distinct concepts, the articulation of the features of the dominant engineering culture and discourse, engineering education paradigm and engineering identity converge towards a similar valorisation of the technical over the social in engineering education. The cultural identity of engineering reflected in the curriculum is of a more rigorous, difficult and complex discipline, a masculine field, fit for those who excel in mathematics and the physical sciences, devoid of subjectivity, and with a low concern towards societal issues (Carberry & Baker, 2018; Cech, 2014; Godfrey & Parker, 2010; Pawley, 2008; Stevens et al., 2007; Stonyer, 2002; Tonso, 1999).

These cultural characteristics of engineering are seen to, on one hand, influence the development of an engineering identity as “nuts and bolts” technicists (Faulkner, 2007), according to which engineers are distinguished as an occupational group in light of their technical and scientific expertise (Trevelyan, 2014; Meiksins, 2007, p. 122), and on the other hand, are reflected in the overemphasis of technical and scientific aspects in the engineering curriculum to the exclusion of ethical and societal concerns (Bucciarelli, 2008; Jamison et al., 2014; Johnston et al., 1996; Stevens et al., 2007). The culture of engineering education appears to promote the dichotomy between “hard” and “soft” skills (Martin, 2020), according to which ethics is a “fuzzy” subject (McGinn, 2003), falling outside the scope of “real engineering” (Polmear et al., 2018) and considered “not very important” or of an “inferior quality” (Lönngren, 2021). Thus, what emerges for the purpose of the present analysis is a collective understanding of what it is to be an engineer and educate an engineer as a key generative mechanism for explaining the state and status of engineering ethics education.

Nevertheless, as Tonso (1996, p. 218) points out, culture is an everchanging system of meaning, which holds the promise for improving engineering education towards more inclusive ways or a broader understanding of the engineer’s societal role. We already witness efforts in this direction, represented by non-mainstream currents in engineering that engage the social and ethical dimensions, evidenced by research in engineering studies and practices like community engagement (Lucena et al, 2010; Schneider et al., 2008), humanitarian engineering (Lucena et al., 2003; Mazzurco & Daniel, 2020), decolonial movements (Cordeiro Cruz, 2021; Kutay et al., 2018) or social justice (Baillie, 2020; Karwat, 2020; Karwat et al., 2015; Larsen & Gärdebo, 2017; Nieusma, 2013; Niles et al., 2020a; Riley, 2008).^7^

## Conclusion and Recommendations

The aim of our analysis was to develop deeper levels for understanding engineering ethics education (Mc Evoy & Richards, 2006, p. 69). We regarded this analysis of the current state and status of engineering ethics education as a prerequisite for suggesting strategies for change towards a socio-technical paradigm of engineering education that could lead to a curricular orientation *for* ethics. Following Wynne (2014, p. 1479), we understand by “orientation” the acceptance of an attitude, of a way of doing things and of operationalising core values.

We argued that engineering ethics education is a complex system, constitutive of various beliefs and practices, which are manifest at different levels. The different levels of engineering ethics education rendered in Table 1 are connected. The analysis showed how the beliefs and practices of individual instructors are impacted by institutional measures and policies set by accrediting bodies, as well as by the cultural milieu in which they were educated or currently teach, while also playing a role in shaping the engineering curriculum. Instructors justify their curricular choices according to their vision of what engineering practice is (Monteiro, 2016; Quinlan, 2002) and their understanding of engineers’ responsibilities (Downey et al., 2007). This has implications for generating change in engineering education, as the instructors’ belief systems influence the diffusion of innovations in engineering education (Boland, 2014; Carew & Mitchell, 2002; Froyd et al., 2008; Quinlan, 2002; Seymour, 2002; Sonnert, 2007; Spalter-Roth & Meiksins, 2008). Thus, change in teaching practices often requires forming new collective identities about what is valued in engineering education (Carberry & Baker, 2018; Godfrey, 2014; Quinlan, 2002).

At the same time, issues related to the purpose of engineering education and the perception of ethics in the engineering curriculum arise at each level. Recalling Snow (1959), the findings of the analysis reveal the existence of two distinct cultures reflected at the surface level of the engineering curriculum, pointing to ethics’ lesser status. As such, ethics has been articulated as a “soft” and “non-essential” feature of engineering education, a curricular “add-on” implemented in a non-systematic manner and surrounded by a degree of confusion as to its conceptualization and application. The development of technical acumen, on the other hand, is regarded as an essential part of engineering education, and is at the centre of curricular design (Goold, 2015; Martin, 2020).

To dismantle the two cultures existing in engineering education, it is imperative to move from a non-essential status given to ethics towards a socio-technical orientation of the engineering curriculum *for* ethics. Engineering education *for* ethics is a transformative process, which aims to challenge existing core assumptions and values promoted in engineering education (Cranton, 2006; Mezirow, 1978, 1991; Sheppard et al., 2009). Although many studies focused on the transformation of higher education, and specifically on higher education *for* sustainability (Filho et al., 2018; Holmberg et al., 2012; Trowler et al., 2013), the question of the integration of the ideal of engineering education *for* ethics has been largely ignored, highlighting a potential area for further research.

Furthermore, it has been remarked that change strategies need to link different levels for generating a long-lasting transformation (Graham, 2012; Hannah & Lester, 2009). When aiming to effect change, it is important to take a systemic rather than a linear approach (Sterling, 2004), which implies thinking “vertically, about interdependencies at higher and lower levels” (Trowler, 2008, pp. 155–157). Our undertaking to identify the different types of agents and forces shaping engineering education is a necessary first step. As Rover (2008, p. 389) notes, the key to change is first understanding “what we are”, and then taking steps towards “what we are capable of becoming”. Building on this, it is imperative to examine the role of each in the socio-technical orientation of engineering education *for* ethics, towards a “hybrid” and “comprehensive” paradigm that integrates the scientific, technical, social, political and environmental dimensions of engineering, as envisioned by Jamison et al (2014) and van den Hoven (2019).

At cultural level, there is a need for determining the different professional identities actively promoted by engineering programmes, as well as the meanings imparted through the ethos fostered in institutions and the structure of the engineering curricula. Patrick and Borrego (2016, p. 4) point out that studies of identity development tend to use a narrow definition and do not give credence to the socio-cultural and environmental factors that shape “becoming” in the process of “doing” engineering. While discussing the factors affecting engineering identity development, Morelock (2017, p. 1250) recalls only one study (Paretti & Mc Nair, 2012) which points to the discourse that challenges or reinforces extant engineering identities as a directional factor shaping the type of engineering identity that students might develop. Following Morelock (2017, p. 1256), we stress the importance for researching engineering identity development to “examine how frameworks that define individual engineering identity harmonise with how societal conditions have shaped collective engineering identity in participants’ national contexts”. A further aspect to be considered is researching the effects of different methods of implementing and teaching ethics on the development of a socio-technical identity of engineering students, resonating with efforts conducted by Johnson et al. (2016), Leidens et al. (2018) and Jesiek et al. (2019). More so, as Nieusma and Cieminski (2018) note, engineering education reformers committed to centering ethics discourse should take a curriculum wide approach focused on the cohesiveness of the diverse components making up students’ educational cultures and not just individualized student knowledge about ethics or capacities for moral reasoning. Achieving this would require bringing to the forefront examples of best practices in centring ethics within the institutional culture, similar to the examples of curricular redesign presented by Riley et al. (2004) and Mitcham and Englehardt (2019).

At policy level, research has revealed the impact of national accrediting bodies on increasing the weight given to ethics in the engineering curriculum (Barry & Ohland, 2012; Lattuca et al., 2006; Skinner et al., 2007). Yet, little is still known on how to maximise the evaluation of ethics in accreditation as to assist programmes in a more systematic implementation (Barry & Ohland, 2012; Bielefeldt et al., 2016; Herkert, 2002; LeBlanc, 2002). Further research is needed for exploring what counts as effective feedback provided by accrediting bodies and how to prepare members of accreditation panels to offer constructive feedback and recommendations targeting the ethical criterion for accreditation.

At the institutional level, upon highlighting the need for a systematic implementation of ethics (Flynn & Barry, 2010; Lambrechts et al., 2013; Murphy et al., 2019; Newberry, 2004; Polmear et al., 2018), it is crucial to research strategies for curriculum redesign and identify examples of best practices in the development of a holistic and comprehensive educational model. Recent years saw growing debates and research on education *for* sustainability (Filho et al., 2018; Holmberg et al., 2012; Trowler et al., 2013) and similar attention should be given to engineering education *for* ethics. Given the limited research available on curricular alignment and quality insurance in engineering ethics education (Bombaerts et al., 2019; Hess & Fore, 2018; Keefer et al., 2014; Li & Fu, 2012; Romkey, 2015), we highlight the need for further research to explore the effectiveness and coherence between the implementation, teaching, assessment methods, the goals and theoretical frameworks envisioned for engineering ethics education. More specifically, research should illuminate ways to ensure curricular alignment between theoretical frameworks, the institutional vision, learning goals, content themes, teaching and assessment methods.

At individual level, we recommend additional research exploring instructors’ understanding of what falls under the scope of engineering ethics education and the goals employed, to illuminate whether ethics instructors adopt any of the various theoretical conceptualisations of ethics developed or whether a common-sense understanding of ethics prevails. In terms of the former, further research could help determine which ethics learning goals are favoured by instructors. This should be complemented by researching the attainment of these goals, given that “ethical awareness has not been demonstrated to translate reliably into ethical behaviour” (Bairaktarova & Woodcock, 2017, p. 1130). As pointed out by Martin et al. (2021) and Bombaerts et al. (2021), metrics of evaluating the effectiveness of different teaching approaches are still underdeveloped.

Given the need for more guidance in engineering ethics instruction, a recommended avenue for further research is to provide an in-depth exploration of the challenges experienced by instructors when teaching and assessing ethics. It is equally important to examine the impact of different strategies in countering these challenges, as well as the role of funding streams dedicated to engineering ethics education research, of independent support initiatives, of repositories such as the Online Ethics Center, or working groups on ethics affiliated with international societies of engineering education such as SEFI or ASEE. The extensive literature that is the object of this review is based in the US, with several empirical studies coming from projects which received the financial support of the National Science Foundation. This seems to point to the importance of a dedicated funding stream for engineering education research with cascading effects on the education of engineering ethics, to be replicated as a policy strategy in other geographical contexts.

Following Kim et al.’s (2018) and Niles et al.’s (2020b) suggestion, further research is also needed to understand why engineering students are disengaged from the societal and public welfare role of engineering, and which strategies can reverse this trend. It is also important to examine the effectiveness of various teaching approaches for enhancing students’ reception of the subject, given that it was identified as a challenge for engineering ethics instructors (Harding et al., 2009; Polmear et al., 2018; Romkey, 2015). Additionally, this might require research targeted at developing curriculum materials, guidelines and textbooks (Reed et al., 2004), consistent with empirical findings on the effectiveness of different teaching methods. A particular focus should be given to empirical research on the design and application of different typology of case studies (Martin et al., 2021), given the popularity of this teaching method (Bagdasarov et al., 2013; Lundeberg, 2008; Abaté, 2011; Romkey, 2015; Thiel et al., 2013; Yadav & Barry, 2009).

An agenda for a socio-technical orientation of engineering education *for* ethics would thus call to:

(i) clarify the underlying paradigm driving the development of engineering education initiatives and programmes,
(ii) reconceptualise what it means to be an engineer and to educate an engineer, for developing a socio-technical professional identity,
(iii) enhance the role of humanities, social sciences, science and technology studies or liberal arts studies in engineering education,
(iv) prepare students to engage with public policy, as to enable an engineering practice committed to human welfare, sustainability and social justice,
(v) generate commitment to larger systematic change to established practices over time, rather than suggest heroic responses to management wrongdoing,
(vi) foster reflection on how the practices of engineers impact and are impacted by their socio-cultural environment and how they can be changed,
(vii) ask how engineering education and society can change together in a mutually affirming way, towards more sustainable patterns for both (Sterling, 2004, p. 67),
(viii) address the organisational, political and socio-economic factors that impinge on engineering practices and provide a theoretical lens for understanding them.

## Footnotes

1 We want to thank Carl Mitcham for the suggestion that engineering might have been considered as morally good.

2 Henceforth, abbreviated as VSD.

3 The authors want to thank the anonymous reviewer #7 for this interpretation.

4 Thank you to Reviewer #7 for suggesting the inclusion of peer support networks.

5 Referring to the cultural dichotomies between natural and social sciences, (Keller, 1985) observes an assumption present in scientific practice between objectivity, reason, and mind, which are cast as male features, and subjectivity, feeling, and nature, which are perceived as female features.

6 Becher & Trowler (2001, p.192) remarked that soft disciplines are “seen internally as politically weak and externally as lacking in good intellectual standing”, which “has rendered the social sciences especially vulnerable to attack from unsympathetic external forces”.

7 We to thank the anonymous reviewer #5 for highlighting the role of current non-mainstream movements in effecting change.
